# Plasma Clot Properties in Patients with Pancreatic Cancer

**DOI:** 10.3390/cancers15164030

**Published:** 2023-08-09

**Authors:** Johannes Thaler, Gerald Prager, Ingrid Pabinger, Cihan Ay

**Affiliations:** 1 Clinical Division of Haematology and Haemostaseology, Department of Medicine I, Medical University of Vienna, 1090 Vienna, Austria; ingrid.pabinger@meduniwien.ac.at (I.P.); cihan.ay@meduniwien.ac.at (C.A.); 2 Clinical Division of Oncology, Department of Medicine I, Medical University of Vienna, 1090 Vienna, Austria; gerald.prager@meduniwien.ac.at

**Keywords:** pancreatic cancer, plasma clot formation, tissue factor

## Abstract

**Simple Summary:**

We detected an increased plasma clot formation rate and an increased delta absorbance (ΔAbs, indicating fibrin fiber thickness) in pancreatic cancer patients compared to sex-matched healthy controls applying a modified plasma clot formation assay, in which only CaCl_2_ and phospholipids were added to initiate clotting. Following addition of a tissue factor blocking antibody in our modified assay, the plasma clot formation rate decreased significantly in patients only, ΔAbs significantly decreased in patients and in healthy controls, the lag phase did not change, and the time to peak fibrin generation increased in patients only. Taken together, these findings indicate the presence of a prothrombotic state in pancreatic cancer patients, which depends on tissue factor.

**Abstract:**

Pancreatic cancer is one of the most prothrombotic malignancies. Plasma clot properties may be altered in patients with pancreatic cancer, and circulating tissue factor (TF) may play an important role. We applied a modified plasma clot formation assay (only CaCl_2_ and phospholipids were added to initiate clotting) and a standard clotting assay (lipidated TF was also added) to investigate whether plasma clot properties are altered in pancreatic cancer patients (n = 40, 23 female) compared to sex-matched healthy controls. The modified assay was also performed in the presence of a TF blocking antibody. With this modified assay, we detected an increased plasma clot formation rate (Vmax) and an increased delta absorbance (ΔAbs, indicating fibrin fiber thickness) in patients compared to controls. These differences were not detected with the standard clotting assay. Following addition of a TF blocking antibody in in our modified assay, Vmax decreased significantly in patients only, ΔAbs significantly decreased in patients and in healthy controls, the lag phase did not change, and the time to peak fibrin generation increased in patients only. Taken together, these findings indicate the presence of a prothrombotic state in pancreatic cancer patients, which depends on TF and is detectable with our modified assay but not with a standard clotting assay.

## 1. Introduction

The final step of the coagulation cascade is the conversion of fibrinogen into fibrin, which leads to plasma clot formation. Plasma clots prevent excessive bleeding after blood vessel injury and provide a scaffold for tissue repair and wound healing [1]. Under physiological conditions, plasma clot formation is restricted to sites of tissue damage by fibrinolytic enzymes and coagulation factor inhibitors [2].

In 1878, Theodor Billroth first observed that tumors are frequently covered with plasma fibrin clots, and he hypothesized that fibrin deposition may contribute to metastasis formation [3]. Consistently, it has been firmly established in subsequent experimental studies that tumor metastasis formation depends on the deposition of fibrin [4,5,6]. In clinical studies, plasma levels of D-dimer, which is a fibrin degradation product, predicted the risk of developing venous thromboembolism in patients with cancer [7,8,9,10].

Pancreatic cancer is one of the most prothrombotic malignancies [11,12,13]. Fibrin was demonstrated to be present on the surface of pancreatic cancer cells and throughout the tumor stroma [14,15]. A central role in the prothrombotic state of pancreatic cancer has been suggested for circulating extracellular vesicles that expose tissue factor, the main initiator of the coagulation cascade. Such tissue-factor-exposing extracellular vesicles are released from pancreatic cancer cells and efficiently convert fibrinogen into fibrin [16,17]. Moreover, we previously reported that extracellular vesicles from the plasma of patients with metastatic pancreatic cancer shorten the clotting time of pooled normal plasma in a tissue-factor-dependent manner [18].

In the present study, we applied a standard plasma clot formation assay [19,20,21,22] and a modification of this assay to investigate whether plasma clot properties are altered in patients with pancreatic cancer compared to sex-matched healthy controls and, if so, whether these alterations depend on tissue factor. We also investigated correlations between plasma clot properties and routine clinical and laboratory parameters.

## 2. Materials and Methods

### 2.1. Study Design and Study Population

The study protocol was approved by the local Ethics Committee in accordance with the Declaration of Helsinki (EK Nr: 404/2009). A total of 40 patients with histologically confirmed primary adenocarcinomas of the pancreas, diagnosed and treated at the Vienna General Hospital, and 40 sex-matched healthy individuals were recruited for this study. The disease stage was classified according to the international TNM staging system.

### 2.2. Blood Sampling

Venous blood samples were drawn into citrate vacuum tubes (Vacuette; Greiner-Bio One, Kremsmuenster, Austria) through atraumatic and sterile antecubital venipuncture on the day of study entry. The citrated blood was centrifuged at 1550× *g* for 20 min to obtain platelet-poor plasma (PPP). The centrifugation of each sample was performed within 1 h after blood sampling, and the freezing of each sample within 1 h after centrifugation. Plasma aliquots were stored at −80 °C until plasma clot formation was measured in series.

### 2.3. Routine Laboratory Parameters

Routine laboratory parameters were determined in the central laboratory of the General Hospital Vienna according to protocols that are implemented in routine clinical practice (www.kimcl.at, accessed on 2 August 2023).

### 2.4. Plasma Clot Formation Assay

Plasma clot formation was measured turbidimetrically as reported previously [20]. Briefly, in the standard plasma clot formation assay, clotting was measured after re-calcification of plasma (CaCl_2,_ 20 mmol/L, final), addition of phospholipids, and addition of recombinant lipidated tissue factor (TF, Innovin, 1 pmol/L, final). In our modified plasma clot formation assay, clotting was measured after the addition of CaCl_2_ and phospholipids (recombinant lipidated TF was omitted). For the detection of plasma clot formation, absorbance was read in duplicates at 405 nm for one hour at 12 s intervals in a SpectraMax 340 Plus plate reader (Molecular Devices, Sunnyvale, CA, USA) in the absence and presence of a TF-blocking antibody (HTF1, 4 μg/mL; BD Biosiences, San Jose, CA, USA) or a control antibody (mouse IgG: 4 μg/mL; Sigma-Aldrich, St. Louis, MO, USA).

**Figure 1:**
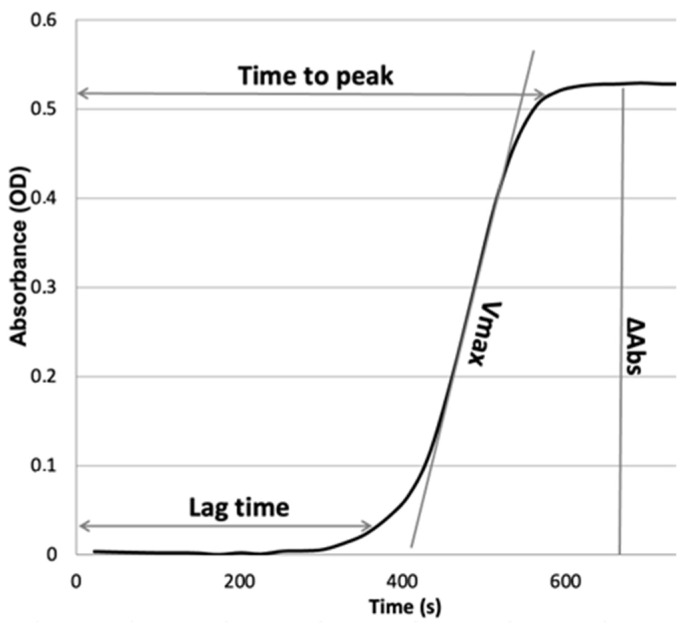
The plasma clot formation curve indicates the dynamics of absorbance at 405 nm over time. All analyzed parameters of plasma clot formation are shown. Lag time, time until the onset of clot formation; Vmax, the maximum rate of turbidity increase; ΔAbs, maximum absorbance at plateau.

The lag phase of the turbidity curve, reflecting the time until the onset of clot formation, was recorded. To assess the rate of fibrin formation, the maximum rate of turbidity increase (Vmax) was read by fitting a line through 5–10 points on the slope. The measured maximum absorbance at plateau (ΔAbs) reflects fiber thickness and structure (Figure 1).

### 2.5. Statistics

Continuous variables were described by the median and the interquartile range (IQR, 25th–75th percentile). Categorical variables were described by the absolute numbers and percentages. The Wilcoxon rank sum test was used for group comparisons. The correlations between variables were assessed using Spearman’s rank correlation coefficients. Two-sided *p*-values smaller than 0.05 were considered as indicating statistical significance. Statistical analyses were performed with SPSS Version 17.0.2.

## 3. Results

### 3.1. Clinical Characteristics and Routine Laboratory Parameters

Clinical characteristics and routine laboratory parameters of pancreatic cancer patients and healthy controls are provided in Table 1. In pancreatic cancer patients, fibrinogen, D-dimer, and CRP values were significantly higher than in healthy controls.

### 3.2. Plasma Clot Formation

Results on plasma clot formation in patients with pancreatic cancer and healthy controls are summarized in Table 2.

### 3.3. Modified Plasma Clot Formation Assay (Clotting Induced by CaCl_2_ and Phospholipids) in the Absence and Presence of a Tissue Factor Blocking Antibody

**Figure 2:**
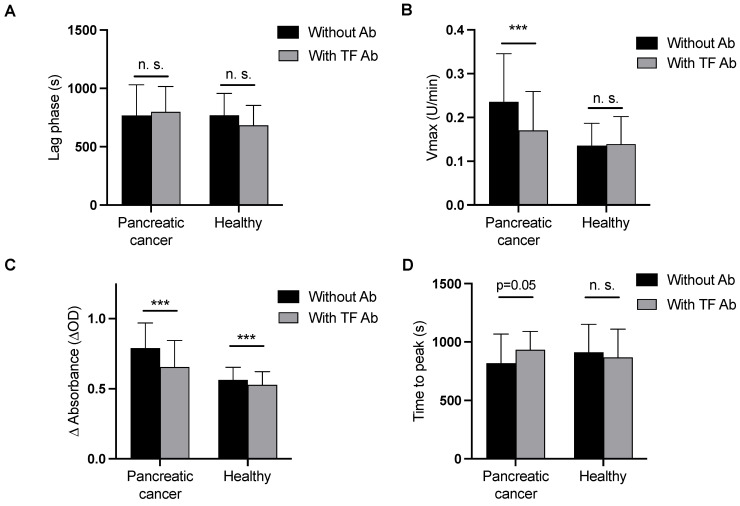
(**A**–**D**) Plasma clot formation initiated by CaCl_2_ and phospholipids (i.e., the modified plasma clot formation assay) with and without a tissue factor blocking antibody (HTF-1) in pancreatic cancer patients and healthy controls, (*** = *p* ≤ 0.001; n.s., not significant).

Results on clot formation induced by CaCl_2_ and phospholipids are provided in Table 2 and Figure 2. The time until clot formation started (lag phase) and the time to peak fibrin generation did not differ between patients and controls. The maximum plasma clot formation rate (Vmax) was significantly increased in pancreatic cancer patients compared to healthy controls. Additionally, the maximum absorbance at plateau (ΔAbs), which reflects fiber thickness, was significantly increased in pancreatic cancer patients compared to controls.

Following addition of a TF blocking antibody, the lag phase did not change in pancreatic cancer patients and healthy controls, Vmax decreased significantly in pancreatic cancer patients but not in controls, ΔAbs decreased significantly in pancreatic cancer patients and in healthy controls, and time to peak fibrin generation was reduced in pancreatic cancer patients with borderline significance, but not in controls (Figure 2).

### 3.4. Modified Plasma Clot Formation Assay According to Tumor Stage and Grade

**Figure 3:**
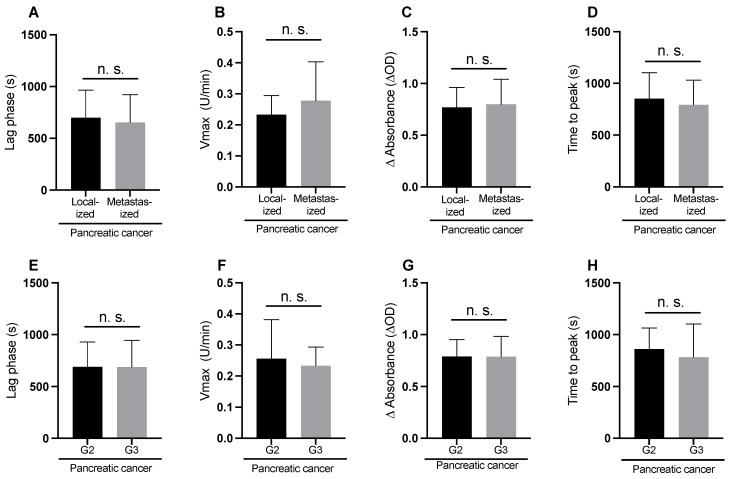
Plasma clot formation in pancreatic cancer patients initiated by CaCl_2_ and phospholipids (i.e., the modified plasma clot formation assay) according to tumor stage (**A**–**D**) and tumor grade (**E**–**H**), n.s., not significant.

Fibrin clot formation parameters did not differ significantly between patients with localized and metastatic tumors and between those with moderately and poorly differentiated tumors (Figure 3A–H).

### 3.5. Standard Clot Formation Assay (Clotting Induced by CaCl_2_, Phospholipids, and Lipidated Tissue Factor)

**Figure 4:**
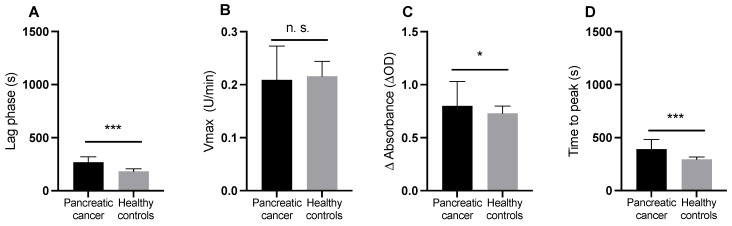
(**A**–**D**) Plasma clot formation initiated by CaCl_2_, phospholipids, and lipidated tissue factor (Innovin) (i.e., the standard plasma clot formation assay) in pancreatic cancer patients and healthy controls. (* = *p* ≤ 0.05; *** = *p* ≤ 0.001; n.s., not significant).

Following addition of CaCl_2_, phospholipids, and lipidated TF no significant difference in Vmax was detected between pancreatic cancer patients and controls. ΔAbs was significantly higher in patients. Lag phase and time to peak fibrin generation were significantly longer in patients that in controls (Table 2, Figure 4A–D).

### 3.6. Correlations between Plasma Clot Properties and Routine Laboratory Parameters

Spearman correlation coefficients between plasma clot parameters, routine coagulation parameters (prothrombin time, activated partial thromboplastin time, fibrinogen, D-dimer, platelet count), inflammatory parameters (leukocyte count and CRP) and the tumor marker Ca19-9 of pancreatic cancer patients are provided in Table 3.

## 4. Discussion

In the current study, we applied a standard plasma clot formation assay and a modification of this assay in pancreatic cancer patients and healthy controls.

In the modified plasma clot formation assay (in which we initiated clotting by addition of CaCl_2_ and phospholipids only), we detected an increased plasma clot formation rate (Vmax) and an increased absorbance at plateau (ΔAbs) in pancreatic cancer patients compared to healthy controls. Vmax and ΔAbs were inhibited by addition of a TF blocking antibody in pancreatic cancer patients. This points to the presence of endogenous coagulant TF in the plasma of pancreatic cancer patients. Interestingly, also in healthy controls, we detected a small but significant decrease in ΔAbs after the addition of a TF antibody, which may point to the presence of low concentrations of functional endogenous TF in plasma of healthy individuals. However, the presence of functional TF in healthy individuals is currently controversially discussed and needs to be further investigated [23]. Applying the standard plasma clot formation assay (in which lipidated TF was also added to initiate clotting) the plasma clot formation rate did not differ between patients and controls. With the standard clot formation assay an increased delta absorbance (indicating fibrin fiber thickness) and a longer lag phase and longer time to peak were observed in pancreatic cancer patients compared to healthy controls. This may be explained by increased fibrinolysis [24], evidenced by elevated D-dimer levels, in pancreatic cancer patients in this study. Taken together, these findings may indicate that that addition of recombinant lipidated TF, which is used in the standard plasma clot formation assay, may obscure the detection of endogenous TF.

In our modified assay, plasma clot formation parameters did not differ between patients with localized and metastatic pancreatic cancer and between patients with moderately (G2) and poorly (G3) differentiated pancreatic cancer (Figure 4). This is in contrast to previous studies that applied a chromogenic factor Xa generation assay, referred to as extracellular vesicle-associated TF activity (EV-TF activity) assay [18,25,26,27]. In these studies, EV-TF activity consistently predicted worse survival outcome, which is a surrogate parameter for advanced tumor stage and high tumor grade [28,29], and EV-TF activity was highly elevated in patients with poorly differentiated metastatic pancreatic cancer [18]. In our modified plasma clot formation assay, we used the same antibody (HTF-1) to block TF activity, as in the aforementioned studies. So why did we not detect differences according to tumor grade and stage? First of all, in our assay, plasma clot formation was measured with minimal pre-analytical modifications of plasma. In contrast, in the previously applied EV-TF activity assay, pre-analytic sample preparation is extensive, and has been shown to impact EV levels and the coagulant potential of EVs [30]. The EV-TF activity assay may also be more sensitive for the detection of pathological TF-exposing EVs, because measurements are performed in absence of natural coagulation inhibitor (i.e., outside the plasma milieu) as EV are pelleted and resuspended in buffer (therefore outside the plasma milieu). In addition, pelletation and resuspension of EVs increases phosphatidylserine exposure on EVs, and thereby may de-crypt nascent TF [30,31,32].

It is a limitation of this study that we did not inhibit contact activation in our plasma samples. As clotting occurred relatively late in our modified clotting assay, a significant contribution of factor XIIa and XIa to plasma clot formation seems likely. In future studies, plasma clot formation measurements in cancer patients should also be performed in presence of an FXII inhibitor (such as corn trypsin inhibitor) or an FXI inhibitor (such as abelacimab) [33,34]. However, the concept of two separate (i.e., intrinsic and extrinsic) coagulation pathways is outdated and has been replaced by the cell-based TF-driven model of coagulation activation [2], and evidence is accumulating that indicates a role for FXII and FXI in the development of venous thromboembolism rather than physiological hemostasis [35]. Calculation of the Khorana score and investigation of correlations with parameters of our clotting assay would have been of interest. However, the most important parameter of the Khorana score for VTE risk stratification is cancer type, and this parameter is of no use in the current population as only pancreatic cancer patients were included. Moreover, pre-chemotherapy laboratory parameters were not available, but these parameters are needed for calculation of the Khorana score.

We also investigated correlations between plasma clot formation and routine laboratory parameters. In our modified assay, Vmax correlated with prothrombin time and with D-dimer levels, and ΔAbs correlated strongly with fibrinogen levels in pancreatic cancer patients. In the standard clot formation assay, correlations between Vmax and fibrinogen, CRP, and tumor marker Ca 19-9 levels were found, and in the standard assay, a strong correlation between ΔAbs and fibrinogen levels was found.

The concrete clinical impact of our findings is currently limited, because our modified clotting assay has not yet been applied by other research groups. A next step would be to perform inter-laboratory comparisons in a multi-center study to investigate the reproducibility of our assay. In addition, we still need to investigate in a prospective study whether specific parameters of our modified clotting assay indeed predict the risk of developing future VTE in pancreatic cancer patients. However, taken together, data from our study provide a solid basis for future studies and further investigations on the role of TF in pancreatic cancer.

## 5. Conclusions

Taken together, our findings indicate the presence of a prothrombotic state in pancreatic cancer patients that depends on TF and is detectable with our modified plasma clot formation assay, but not with a standard clotting assay.

## Figures and Tables

**Table 1:** Baseline characteristics and routine laboratory parameters of pancreatic cancer patients and healthy controls.

	Pancreatic Cancer	Healthy	*p*-Value
**Basic Clinical Characteristics**			
Number, n	40	40	n.s.
Sex, female, n (%)	23 (58%)	23 (58%)	n.s.
Age, years	66 (56–72)	55 (44–62)	<0.001
Tumor stage, n (%)	Localized: 21 (53%) Metastatic: 19 (47%)	-	-
Tumor differentiation, n (%)	High (G1): 0 Moderate (G2): 26 (65%) Poor (G3): 14 (35%)	-	-
Comorbidities, n (%)	Hypertension: 22 (55%) Diabetes: 11 (27.5%) Coronary heart disease: 4 (10%) COPD: 2 (5%)	-	-
Anticoagulation with low molecular weight heparin, n (%)	Prophylactic: 6 (15%) Therapeutic: 0		
**Routine laboratory parameters** (median, 25th–75th percentile)			
Prothrombin time, %	86 (76–112)	92 (83–104)	n.s.
aPTT, s	33.9 (31.8–38.9)	34.5 (32.8–36.3)	n.s.
Fibrinogen, µg/mL	418 (366–491)	306 (264–352)	<0.001
D-dimer, µg/ml	1.38 (0.75–2.90)	0.26 (0.26–0.41)	<0.001
Platelet count, 10^9^/L	269 (212–348)	274 (219–297)	n.s.
Leukocyte count (×10^9^/L)	6.32 (5.02–8.50)	6.26(5.43–6.94)	n.s.
CRP (mg/dL)	1.57 (0.34–2.96)	0.10 (0.06–0.23)	<0.001
Ca 19-9 (U/mL)	235.5 (30.6–1102)	-	-

**Table 2:** Plasma clot properties of pancreatic cancer patients and sex-matched healthy controls.

	Pancreatic Cancer	Healthy	*p*-Value
**Plasma clot formation parameters (median, 25th–75th percentile)**			
**- Modified plasma clot formation assay** (addition of CaCl_2_ + phospholipids)			
Lag phase (s)	688 (606–925)	736 (629–895)	n.s.
Vmax (U/min)	0.235 (0.170–0.346)	0.136 (0.116–0.185)	<0.001
ΔAbs (ΔOD)	0.789 (0.609–0.968)	0.563 (0.450–0.652)	<0.001
TTP (s)	818 (726–1065)	912 (741–1148)	n.s.
**- Modified plasma clot formation assay + anti tissue factor antibody**			
Lag phase (s)	799 (660–987)	683 (550–853)	n.s.
Vmax (U/min)	0.170 (0.132–0.259)	0.139 (0.109–0.201)	0.006
ΔAbs (ΔOD)	0.654 (0.541–0.840)	0.528 (0.443–0.616)	0.001
TTP (s)	933 (852–1088)	867 (711–1107)	n.s.
**- Standard plasma clot formation assay** (addition of CaCl_2_ + phospholipids + lipidated tissue factor)			
Lag phase (s)	267 (235–319)	182 (162–206)	<0.001
Vmax (U/min)	0.209 (0.151–0.273)	0.216 (0.163–0.237)	0.86
ΔAbs (ΔOD)	0.801 (0.685–0.103)	0.730 (0.589–0.794)	0.03
TTP (s)	390 (340–482)	294 (260–314)	<0.001

**Table 3:** Spearman correlation coefficients between plasma clot properties and routine laboratory parameters in patients with pancreatic cancer.

	(A) Modified Plasma Clot Formation Assay				(B) Modified Plasma Clot Formation Assay + Tissue Factor Blocking Antibody				(C) Standard Plasma Clot Formation Assay			
	Vmax	ΔAbs	Lag Phase	Time to Peak	Vmax	ΔAbs	Lag Phase	Time to Peak	Vmax	ΔAbs	Lag Phase	Time to Peak
**Prothrombin time, %**	0.359 *	0.362 *	−0.041	0.071	0.434 *	0.261	−0.101	−0.018	0.322	0.285	−0.211	−0.78
**aPTT, s**	0.108	−0.114	0.019	0.002	−0.123	−0.145	−0.097	−0.090	−0.0105	−0.150	−0.064	0.152
**Fibrinogen, mg/dL**	0.126	0.541 **	0.307	0.255	0.326	0.605 **	0.128	0.112	0.597 ***	0.768 ***	0.170	0.144
**D-dimer, μg/mL**	0.395 *	0.164	−0.262	−0.226	0.318 *	0.145	−0.192	−0.334 *	0.042	−0.045	0.274	0.206
**Platelet count, G/L**	0.049	0.119	0.081	0.020	−0.134	0.104	0.035	−0.013	0.131	0.190	0.052	0.124
**Leukocyte count (×10^9^/L)**	0.276	0.037	−0.130	−0.280	0.086	−0.091	−0.071	−0.197	−0.006	0.321	0.207	0.239
**CRP (mg/dL)**	0.350	0.217	−0.121	−0.103	0.313	0.217	−0.071	−0.344	0.411 *	0.223	0.143	0.092
**Ca 19-9**	0.097	0.102	0.071	0.033	−0.065	0.095	−0.059	0.056	0.325 *	0.093	0.089	−0.082

* *p* ≤ 0.05, ** *p* ≤ 0.01, *** *p* ≤ 0.001.

## Data Availability

The data presented in this study are available on request from the corresponding author.
